# Towards a Treatment of Stress Urinary Incontinence: Application of Mesenchymal Stromal Cells for Regeneration of the Sphincter Muscle

**DOI:** 10.3390/jcm3010197

**Published:** 2014-02-24

**Authors:** Wilhelm K. Aicher, Melanie L. Hart, Jan Stallkamp, Mario Klünder, Michael Ederer, Oliver Sawodny, Martin Vaegler, Bastian Amend, Karl D. Sievert, Arnulf Stenzl

**Affiliations:** 1 KFO273, Department of Urology, University of Tuebingen Hospital, Tuebingen 72076, Germany; E-Mails: melaniehar@googlemail.com (M.L.H.); martin.vaegler@med.uni-tuebingen.de (M.V.); bastian.amend@med.uni-tuebingen.de (B.A.); karl.sievert@med.uni-tuebingen.de (K.D.S.); arnulf.stenzl@med.uni-tuebingen.de (A.S.); 2 FRAUNHOFER Institute, Klinikum Mannhein, Mannheim 68167, Germany; E-Mail: jan.stallkamp@ipa.fraunhofer.de; 3 Department for Systems Dynamics, University of Stuttgart, Stuttgart 70569, Germany; E-Mails: mario.kluender@isys.uni-stuttgart.de (M.K.); michael.ederer@isys.uni-stuttgart.de (M.E.); oliver.sawodny@isys.uni-stuttgart.de (O.S.); 4 Department of Urology, University of Tuebingen Hospital, Tuebingen 72076, Germany

**Keywords:** mesenchymal stromal cells, mesenchymal stem cells, urinary stress incontinence, stem cell application, urodynamics, cell injection techniques

## Abstract

Stress urinary incontinence is a significant social, medical, and economic problem. It is caused, at least in part, by degeneration of the sphincter muscle controlling the tightness of the urinary bladder. This muscular degeneration is characterized by a loss of muscle cells and a surplus of a fibrous connective tissue. In Western countries approximately 15% of all females and 10% of males are affected. The incidence is significantly higher among senior citizens, and more than 25% of the elderly suffer from incontinence. When other therapies, such as physical exercise, pharmacological intervention, or electrophysiological stimulation of the sphincter fail to improve the patient’s conditions, a cell-based therapy may improve the function of the sphincter muscle. Here, we briefly summarize current knowledge on stem cells suitable for therapy of urinary incontinence: mesenchymal stromal cells, urine-derived stem cells, and muscle-derived satellite cells. In addition, we report on ways to improve techniques for surgical navigation, injection of cells in the sphincter muscle, sensors for evaluation of post-treatment therapeutic outcome, and perspectives derived from recent pre-clinical studies.

## 1. Introduction

Stress urinary incontinence (SUI) is not only a major hindrance for any individual affected, but it is a very large social, medical, and economic burden to society [1]. SUI is associated with multifactorial pathologies [2,3], including structural changes in the muscle’s composition, loss of the muscle cells, a surplus of the collagenous fibrous connective tissue, changes in vasculature or enervation, and mechanical load. Current treatment strategies involve muscular training, electrophysiological stimulation, and pharmacological interventions to improve neural activation of the sphincter in female patients. For women, other therapeutic options include mechanical support of the urethra to increase sphincter function but require surgical application of slings or injection of bulking agents. Another problem of treatment of SUI with slings is that it can lead to complications, especially in the long term, and has raised concerns against this type of therapy [4]. Comparably, injection of bulking agents in the urinary sphincter muscle did not meet clinical expectations, although some benefit was noted [5]. For men, artificial urinary sphincter devices were introduced in the early seventies, more than forty years ago [6]. Although some success in treatment of iatrogenic male incontinence after prostatectomy was noted, the design of this type of product and of other mechanical prostheses are repeatedly changed, improved and re-designed, due to notable complications or insufficient long term treatment [7]. Therefore, one can conclude that although there is some improvement for the patients with current treatment regimens, they do not treat the cause of the disease: malfunction of the sphincter complex.

Pre-clinical studies of SUI with stem/stromal cells or progenitor cells have yielded promising results [8,9,10,11,12,13,14,15] suggesting that a cell-based regimen could potentially treat the cause of SUI for some patients. Although experts suggest that cell-based therapies for treatment of SUI are at an experimental stage [16,17,18], recently, several centers reported results on clinical trials [19,20,21,22,23,24,25] using different types of cells including muscle derived stem cells [19,21,25], mesenchymal stromal cells (MSC) [24,26], or myoblasts and fibroblasts [23]. Some of these trials did not meet the quality measures expected from proof-of-principal studies and were retracted [24,27]. However, overall, based on clinical trials [21,22] and positive results from pre-clinical studies involving satellite cells or adipose-derived MSC [11,12,13,14,15], recently a clinical phase II study was initiated [26]. However, it is still unclear what the best approach to cellular therapy for treatment of SUI is. In order to explore the potential of any cell-based therapies for treatment of SUI, it is critical to: (i) Define the optimal type of cell required for regeneration of the sphincter muscle; (ii) Develop gentle but precise surgical techniques to apply the cells without interfering with its already weakened function; and (iii) Improve strategies for exactly determining if the muscular function has improved during follow-up treatment.

Here, we discuss three different types of cells: bone marrow-derived mesenchymal stromal cells, urine-derived stem cells, and muscle-derived satellite cells, and their potential to strengthen the urinary sphincter muscle. In addition we briefly report on specific techniques for surgical navigation and stem cell injection methods, as well as on the development of sensors for monitoring the sphincter muscle post-stem cell treatment, and on a pre-clinical model system.

## 2. Results and Discussion

### 2.1. Autologous Progenitor Cells

#### 2.1.1. Mesenchymal Stromal Cells

Mesenchymal stromal cells (MSC), also called multipotent stromal cells or mesenchymal stem cells, were described for the first time by Friedenstein and colleagues some 40 years ago as fibroblast precursors isolated from mouse bone marrow [28]. Later, MSCs from bone marrow were described as osteogenic precursors [29,30,31,32] and, in 1999, the multi-lineage differentiation capacity of human bone marrow-derived MSC (bmMSC) was reported [33]. MSCs were also detected in adipose tissue [34], term placenta [35], and other sources [36,37]. MSCs modulate immune responses [38,39,40,41], and may serve as a source for growth factors during wound healing [42,43] and tissue regeneration [43,44]. The regenerative potential of MSC was investigated in different tissues, including bone [45], cartilage [46], cardiac tissue [47,48], and MSCs even facilitated outgrowth of neurons [49]. The success reported with MSC applications in the above mentioned studies motivated research to utilize MSC from adipose tissue (called adipose-derived stem cells, ADSC) [14,15] or from bone marrow [50,51,52,53,54] for treatment of urinary incontinence in pre-clinical *in vivo* studies [14,15,51,52]. Post-treatment assessments in these feasibility studies ranged from one week [52] to 13 weeks [53], included different types of cells (ADSC or bmMSC), and different models of incontinence [9]. Therefore, comparison of results reported in these studies must be interpreted with care. However, overall, the application of mesenchymal stromal cells in animal models suffering from experimentally induced urinary incontinence seemed to be beneficial. However, larger and randomized cohorts, and longer follow-up are required to acquire a clearer picture on the risk *versus* benefit ratio.

**Figure 1:**
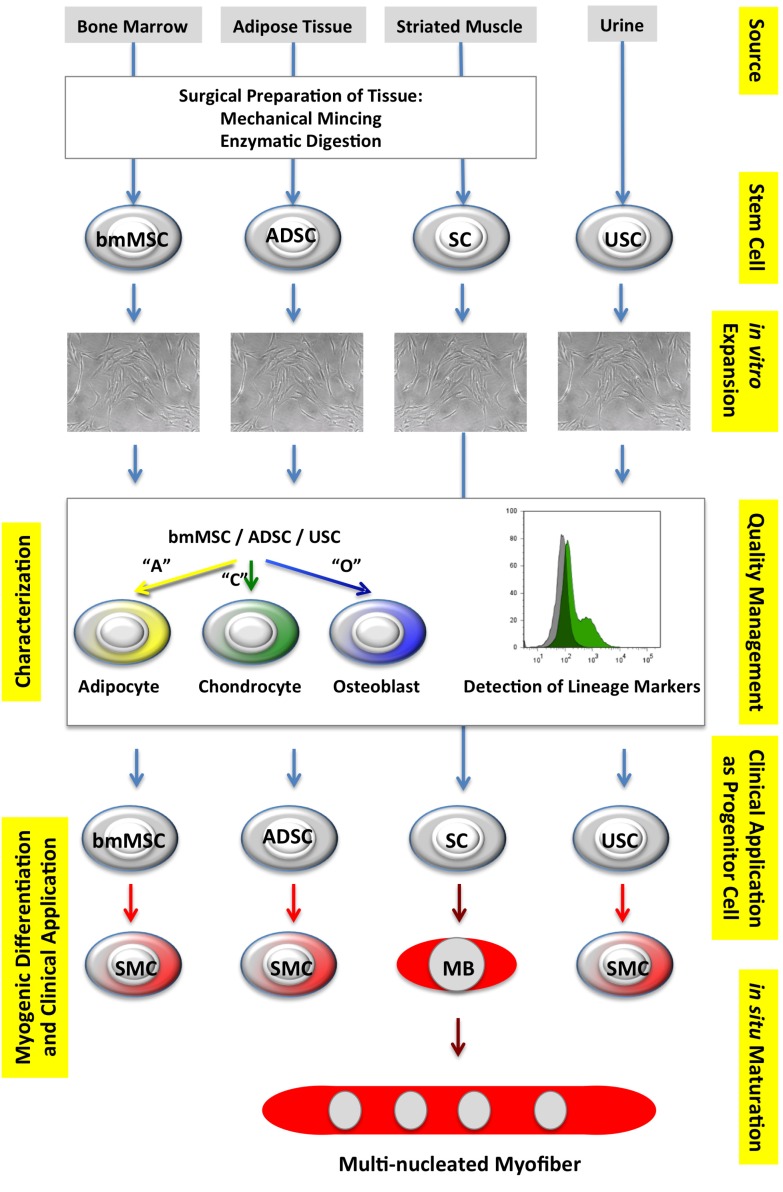
Selected progenitor cells, sources, and myogenic differentiation. Suitable progenitor cells, sometimes also referred to as “stem” cells, can be isolated from different sources, such as bone marrow, adipose tissue, striated muscle, or urine, to generate bone marrow-derived mesenchymal stromal cells (bmMSC), adipose-derived stem cells (ADSC), satellite cells (SC), or urine-derived stem cells (USC). After a primary expansion the quality of bmMSC, ADSC, and USC is explored by adipogenic (“A”), chondrogenic (“C”) and osteogenic (“O”) differentiation of the stem cells, and by detection of the expression of the inclusion/exclusion cell surface antigens [34,37,55]. For SC expression of lineage-specific marker antigens is investigated (not shown). The bmMSC, ADSC, SC, or USC are then either applied as progenitor cells, or incubated in differentiation media to generate smooth muscle cells (SMC), for example, from bmMSC, ADSC or USC, or myoblasts (MB) and multinucleated myofibres from SC.

In terms of the cell types used, application of ADSC or bmMSC may yield several advantages over therapies with muscular progenitor cells including satellite cells or myoblasts in this clinical context: Autologous ADSC or bmMSC can be obtained without intolerable side effects, in sufficient numbers and with an adequate cell quality from patients suffering from SUI (Table 1). Furthermore, bmMSC or ADSC may be applied as undifferentiated progenitor cells, or after myogenic differentiation, *in vitro* (Figure 1). However, the efficacy of myogenic differentiation of both, bmMSC and ADSC in either smooth or striated muscle (like) cells under GMP-compliant conditions, is not yet state-of-the-art. 

**Table 1:** Selected features of human stem or progenitor cells suitable for regeneration of the urinary sphincter in the context cell based therapies for stress urinary incontinence (* SMC: smooth muscle cell).

Stem cell source	Bone marrow	Adipose tissue	Urine	Striated muscle
Cell Type	MSC	ADSC	USC	satellite cell
Key Surface	CD73, CD90	CD34, CD73	CD44, CD73	α7β1 integrin
Inclusion Marker(s)	CD105, CD146	CD90, CD105	CD90, CD105	
Key Surface	CD11b, CD14	CD11b, CD14	CD31, CD34	ø
Exclusion Marker(s)	CD34, CD45	CD45	CD45	
Key Intracellular	vimentin, αSMA	STRO-1	unknown	Pax7
Marker(s)	STRO-1			
Cell Availability	abundant	abundant	abundant	limited
Isolation/Preparation	simple	feasible	very simple	complex
Differentiation	osteo, chondro	osteo, chondro	osteo, chondro	myoblast
Capacities	adipo, SMC *	adipo, SMC *	adipo, SMC *	myotube
		endothelial		
		urothelial		
Smooth Muscle Differentiation	established	established	established	ø
Striated Muscle Differentiation	complex	questionable	published	published
			but not confirmed	
Mode of Action	paracrine/trophic	paracrine/trophic	paracrine/trophic	generation of
	SMC generation	SMC generation	SMC generation	striated muscle cells
Target in SUI	lissosphincter	lissosphincter	lissosphincter	rhabdosphincter
Key References	[15,33,55]	[34,56]	[57,58]	[59,60]

Another benefit of MSC is that, depending on the numbers of cells to be injected (also see Section 2.2.), only a short period of time for expansion MSC is required. Routinely, approximately 1 × 10^7^ total mononuclear cells can be obtained from an average bone marrow aspirate (mean 18 ± 3 mL). This will yield more than 10^7^ proliferation- and differentiation-competent MSC in less than two weeks of *in vitro* culture. For clinical application, these cells have to be expanded under GMP-compliant conditions [61,62]. Comparably, from approximately 6 g of human subcutaneous adipose tissue, approximately 1 × 10^5^–1 × 10^6^ ADSC can be obtained in one week of *in vitro* cultivation. In some cases, MSC derived from term placenta (pMSC) might be an interesting alternative cell source [35]. Within two to three weeks of expansion, about 1 × 10^7^ pMSC can be generated from 100 g of human term placenta. As childbirth is a significant risk factor for SUI, application of autologous MSC from the endometrial part of the term placenta might eventually become a preventive SUI regimen [63]. Therefore, the regenerative potential of MSC from human term placenta has been investigated in more detail, recently, as well [35,41,64,65].

#### 2.1.2. Urine-Derived Stem Cells

Of late, a seemingly unusual source of progenitor cells was described and its potential for regenerative regimens was explored [57,58,66,67,68,69]. Collecting urine samples over three consecutive days from adult males enabled researchers to investigate several protocols for harvesting urine-derived stem cells (USC), their preservation and storage, *in vitro* culture, and characterization and differentiation capacity to generate mature cells with urothel-like and a smooth-muscle like phenotypes [57]. *Ex vivo*, human USC express a panel of cell surface markers such as CD44, CD73, CD90, and CD105, but lacked CD31, CD34, and CD45, suggesting that these cells are of mesenchymal origin and seem closely related to MSC including ADSC (Table 1). CD73 and CD90 were found on preserved USC as well [57]. USC incubated in differentiation media enriched with transforming growth factor-β (TGF-β) plus platelet-derived growth factor-BB (PDGF-BB) induced smooth muscle cell differentiation (Figure 1) as shown by expression of desmin, calponin, smoothelin, myosin, and α-smooth muscle actin [57]. In addition to generation of smooth muscle-like cells *in vitro* [57], USC were also tested for their *in vivo* regeneration capacities and the generation of endothelial cells (as investigated by expression of CD31 and von Willebrand factor) and differentiation to striated muscle-like cells (as explored by detection of desmin, MyoD, and Myf-5) [58]. 

Furthermore, incubation of USC with epidermal growth factor (EGF) induced urothelial cells that expressed the urothelial markers uroplacin, cytokeratins-7, -13, and -20, and the epithelial antigens cingulin and E-cadherin [57]. As the USCs were cloned prior to induction of differentiation, the USCs may be capable of differentiation along two distinct cellular lineages: (i) The mesenchymal lineage of cells, derived from the mesoderm; and (ii) The epithelial cell lineage, derived from the endoderm. However, such an adult progenitor cell plasticity or the trans-differentiation of adult somatic cells across germ line borders are a matter of debate [70], and some people would argue that, at least in bulk USC preparations, stem cells from different germ lines are collected in the starting samples, enabling preferred outgrowth of either the mesenchymal/muscular or the epithelial/urothelial cells. However, by all means, this rather academic discussion distracts from the clinical potential of USC. If applied successfully to ameliorate urinary incontinence in pre-clinical studies, USC may be an interesting alternative to bmMSC, ADSC, or satellite cells (see below) in future urological or gynecological regenerative medicine.

#### 2.1.3. Muscle-Derived Satellite Cells

The omega-shaped urinary sphincter muscle consists of smooth-muscle and striated muscle fibers [71]. Therefore, regeneration and/or performance of both parts of this muscle may be required to gain efficient and long lasting sphincter function. Differentiation of functional striated muscle cells from ADSC or MSC has been reported [14,50,72,73,74]. However, the efficacy in production of *bona fide* striated muscle cells from MSC *in vitro* is rather low. Additionally, the differentiation protocols presented to the scientific public so far either employ recombinant techniques [73], xenobiotic serum or growth factors in the media [72,73], cancerous components [14,50,52], or combinations thereof, to induce myogenic differentiation. A probably safer way to generate striated muscle tissue from progenitor cells may be through the isolation and expansion of satellite cells (SC), as these cells are the natural precursors for generation and regeneration of striated muscle tissue [59,60,75,76]. Satellite cells reside between the basal lamina and sarcolemma in muscle fibers of striated muscle tissue and are activated on demand by growth factors or other stimuli including hypoxia or injury to generate multinucleated myofibers. Therefore, the potential of satellite cells or muscle-derived cells for treatment of SUI has been explored in pre-clinical [11,77,78,79] and even clinical pilot studies [19,21,23,80]. In a study for management of male urinary incontinence, 46% of patients treated with autologous muscle-derived cells did not show improvement, but adverse effects were not at all observed [25]. However, in this study the patients received a mixed cell population, and only approximately 50% of the cells injected expressed typical muscular marker genes [25]. Follow-up in the clinical studies employing “muscular progenitor cells” ranging from one to four years also showed promising results [21,80]. However, failure of therapy in some of these cases may be associated with the individual blend or unclear cellular phenotypes of the cells injected. However, experts agree that striated muscle-derived stem cells or satellite cells do yield clinical benefit for patients suffering from SUI or other forms of incontinence. However, large numbers of satellite cells are required for the injection of cells for the treatment of incontinence. This may result in a severe impact on the healthy muscle at sites where satellite cells are harvested. Therefore some protocols for cell-based treatment of incontinence favor the use of stem or progenitor cells from other sources that yield less side effects, such as bone marrow or adipose tissue. Accordingly, the currently ongoing clinical phase II trial HULPURO employs autologous adipose tissue derived MSC [26], but not satellite cells (Table 1).

### 2.2. Navigation and Improved Cell Application Techniques

The typical steps for cell therapy include the production of the cells, cell implantation, and, finally, following and measuring the treatment’s results. A general trend towards minimally invasive surgery is preferred and, therefore, surgeries are often performed by aid of an endoscope and other high-end imaging instruments [19,25]. Therewith, cell implantation in the urethral sphincter can be performed by a needle/syringe, which is manually pushed through an endoscope to the site of cell injection. From a technical point of view, this procedure using a linearly guided needle is fairly straightforward. However, accurate control of the position of cell injection and distribution of the cells within the sphincter muscle appears a demanding challenge. Furthermore, using needles for injection of cells inherits the risk of tissue damage, possibly accompanied by bleeding.

Based on existing navigation systems for sphincter cell therapy, current research and development are being dedicated to develop novel navigation and cell injection methods for a more precise and less invasive treatment. The workflow for future treatments may look as follows: First, the exact individual anatomical conditions of the patient to be treated and the exact area for injections of the cells in the sphincter are recorded and marked out by MRI and ultrasound imaging (USI) to generate a virtual three dimensional “map”. During treatment, the exact position of the instruments can be visualized as an overlay of the endoscope image, and the “map” generated by the MRI, fused in real-time with the topical USI data. With a tracking system attached to the endoscope and the injection needle, the delicate sphincter muscle can be located and cells can be injected with high precision.

Although optimal positioning of the injection needle is very important, a second aspect of cellular application in the urethral sphincter may be critical for the overall success of such a regimen as well: Optimal spatial distribution of the cells in the tissue itself. Thus far, this has not yet been thoroughly investigated for sphincter regeneration [81]. This is probably due to the fact that many pre-clinical studies employed rodents [11,14,15,51,52,53,54,77,82,83]. In such models, controlled studies comparing, e.g., injection of cells in one site of the sphincter *versus* several injections at different angles or positions, cell dose escalation studies [81], or combinations of cells and biomaterials cannot be addressed due to the anatomical size limitations in these animals. Therefore, these surgical aspects need to be addressed in larger animal models [84]. In addition, a gentle, yet not penetrative, cell injection technology might be helpful as well. In humans, the sphincter muscles measures only a few millimeters in width and thickness [71]. A needle may easily cut through the tissue and cause the cells to be delivered into the peritoneal space. To overcome this limitation of cell injections by needles, new cell application technologies may facilitate the cell application and, at the same time, improve clinical outcome. For example, one possible method would be to “shoot” cells through an endoscopic air pressure nozzle into the tissue. Variation of pressure and volume of the air impulse, design of duct, droplet size, and cell density in the injection fluid may allow to optimization of depth, density, and distribution of the injected cells.

### 2.3. Signal Processing to Evaluate the Regeneration of the Urinary Sphincter Muscle *in Situ*

An objective and reproducible measurement of the sphincter muscle strength is crucial for the assessment of the overall effect of cell-based therapy. Further, a spatially resolved reconstruction of the sphincter muscle strength has the potential to allow the location of weak spots and facilitate the targeted application of stem cells. Currently, no methods are available to measure the spatial distribution of the sphincter muscle strength. Studies investigating the leak point pressure, which can be interpreted as sphincter muscles strength, and its connection to incontinence, exist [85]. However, as leak point pressure is only measured via an intravesical catheter, no data exists on the spatial distribution of the sphincter muscle strength. A novel approach, based on a mathematical model of the urethra and microtip catheter, may overcome this problem. Urethral pressure profilometry is a common tool in the diagnosis for urinary incontinence and can deliver data on the state of the sphincter muscle. To this end, a special catheter is inserted into the urethra and, while it is being slowly retracted, the pressure along the urethra is measured. Several types of catheters capable of measuring pressure inside the urethra are known [86]. One type, the microtip catheter, uses electric pressure transducers on the circumference to measure the local pressure exerted by the sphincter muscle through the urethra on the catheter. Usually, up to four pressure sensors are placed on the catheter’s circumference. However, raw data obtained by those catheters is often difficult to interpret diagnostically. Sensor noise may obscure details and possible angular fluctuations in the pressure profile may not be detected due to limited angular resolution of the sensor arrangement and gaps between the sensors. Additionally, different pressure levels were measured in different directions inside the urethra, the cause of which is still unclear [87]. As those differences cannot be explained through direction-dependent fluctuations of the muscle’s strength, one might have to consider them as artifacts caused by bending of the catheter [88]. An alternative to this approach may be to process the measured data through a mathematical model of the urethra to obtain the pressure profile on the outside of the urethra, which is exerted by the sphincter muscle. This approach can also measure different pressure levels in several directions inside the urethra. 

The first approach to a mathematical model is to describe the urethra as a linear-elastic isotropic hollow cylinder. The system of partial differential equations governing this problem is solved numerically. Therefore the model is discretized through the Finite-Element-Method (FEM). In order to solve this problem, boundary conditions have to be defined. As the catheter is placed inside the urethra, both pressure profile and displacement of the inner boundary are known. However, no information on the pressure profile and displacement on the outside is available. This leads to a so-called inverse problem, which violates at least one condition for a well-posed problem [89]. That means that the solution of this problem is numerically unstable, *i.e.*, tiny variations in the input data cause arbitrarily large variations in the results. Therefore, special algorithms, which are able to recover stability of the solution, have to be developed to solve this problem.

Inverse elasticity problems have been studied extensively in literature [90]. They have been used in some medical applications, including for instance elastography of artery walls [91,92,93]. However, to the best of the authors’ knowledge, inverse algorithms have not been used in urodynamics. Therefore, we propose a mathematical model and an inverse algorithm to reconstruct the spatial pressure profile on the outside of the urethra from measured pressure on the inside. Furthermore, due to the limited angular resolution of the sensor arrangement, data is not continuously available on the inside of the urethra. Hence, sensor positions and areal sensitivity characteristics have to be taken explicitly into account.

**Figure 2:**
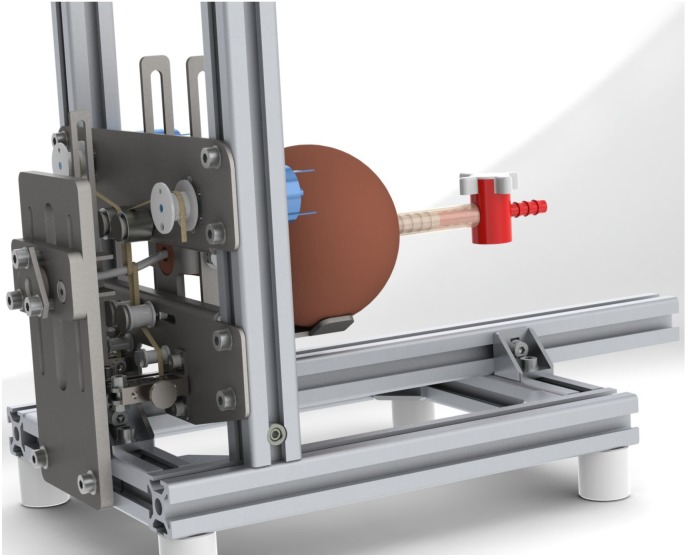
CAD-Rendering of the test stand.

In a second approach, experimental data can be used to test and validate the developed algorithms. Data is obtained for instance on a special test stand, which allows data collection under comparatively well-known and reproducible conditions (Figure 2). Measurements can be conducted with a custom-made microtip catheter with eight pressure sensors on the circumference allowing at the least double the angular resolution compared to standard devices. The test stand mechanically resembles the female urethra. A soft silicone tube emulates the urethra. Strings of different diameters can be wound around the tube in various configurations. They are tightened by electric motors and emulate the sphincter. With this setup, different pressure profiles can be created on the outside of the tube and measured by the catheter.

**Figure 3:**
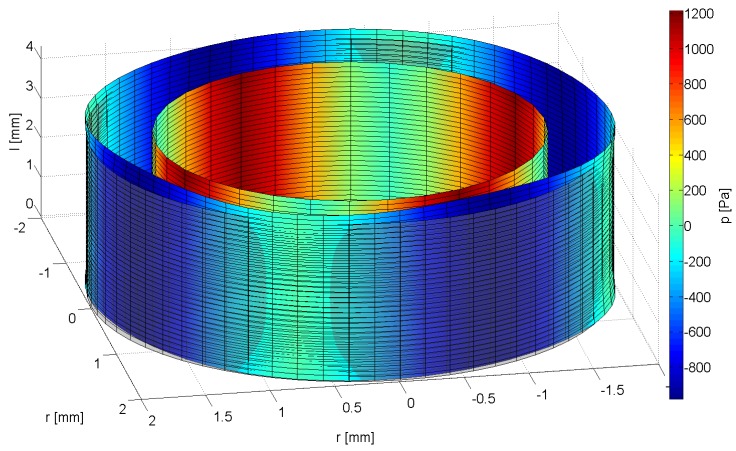
3D-Representation of the deformed urethra model under pressure.

The results of the inverse algorithm are post-processed to three-dimensional figures depicting the deformed shape of the urethra (Figure 3). A color map indicates the pressure level at any given point on the surface. With this method, physicians can easily pinpoint abnormalities and identify differences between before and after the stem cell treatment. 

### 2.4. Pre-Clinical Model

**Figure 4:**
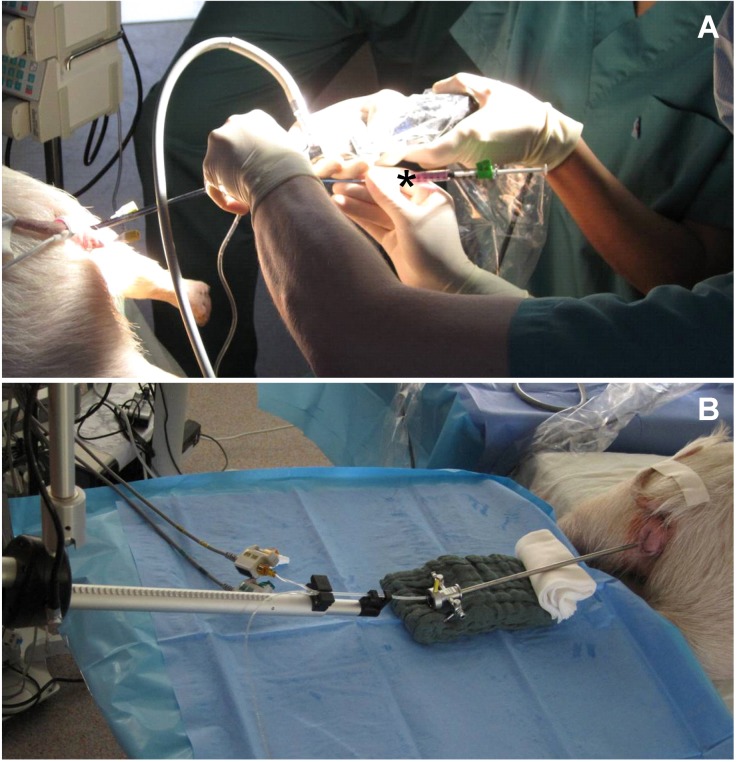
Cell application and functional read-out*.* (**A**) Transurethral injection of cells during cystoscopy procedure under visual control. Defined volumes of labeled cell suspension can be applied via a syringe (asterisk) and needle; (**B**) Urethral pressure profiles (UPP) can continuously be measured by a balloon catheter automatically retracted from the bladder to the rhabdosphincter and urethra.

Many of the currently used reconstructive techniques for treatment of SUI at this point mainly cure the symptoms, instead of functionally regenerating the tissue [94]. Application of regenerative or even differentiated cells directly into the urethral sphincter muscle could potentially recover the function of the sphincter muscle [26]. As tissue samples from patients treated for SUI with cells cannot be harvested without a risk to harm the injected sphincter muscle, mechanisms of such regenerative regimen have to be explored in detail in animal models, such as for instance minipigs (Figure 4). In large animal models the cells can be injected under visual control into the urethral wall using standard clinical instruments. In addition, large animal model allow a comparison of different patterns of cell injections: Cell injections in one site *versus* injections in several sites [25], variation of numbers of cells, and injection volume. The cells can be labeled by paramagnetic particles [95], fluorescent dyes [96], or by recombinant techniques prior to the injection [97]. This enables the investigators to follow the cells after injection in life animals and to localize them by MRI, and visualize the cells *in vivo*. In addition, cells labeled with fluorescent dyes can be localized easily *ex vivo* in tissue samples by microscopy. Moreover, due to the anatomical features of this model, urethra pressure profiles (UPP) in treated *vs.* untreated animals can be monitored again by standard clinical instruments to measure the urodynamic function. 

The goal of pre-clinical studies for cell based sphincter therapies is to develop safe and precise injection technologies and to investigate the behavior and fate of the cells injected. To come as close as possible to the real clinical situation one can even use human cells in a suitable animal model. This however may need some sort of immunosuppression or an immuno-deficient recipient [98]. Based on the hypothesis that human MSC may facilitate endogenous repair mechanisms of porcine sphincter muscles, regimens for mild yet sufficient immuno-suppression were developed. Thus, precise intramuscular application of undifferentiated human MSC for example into the porcine rhabdosphincter may lead to the induction of myogenic *in vivo* differentiation of the cells injected, or result in activation of resident repair mechanisms. This problem—MSC as regeneration promoting cells [43,99] or their functional integration by *in vivo* differentiation [10,54]—represents one of main challenges in realizing this xenogenic transplantation animal model. Moreover, assessing and controlling the fate of the cells after injection is an additional task for quality management and monitoring of the treatment. Achieving these objectives, together with the translation of the entire procedure to clinical routine therapy, will bring this stem cell-based regenerative therapy closer to patients suffering from incontinence compared to any other approach attempted before.

## 3. Conclusions

Mechanical devices are not yet a satisfactory solution to treat stress urinary incontinence. Due to the demographic changes in Western societies, the incidence and prevalence of SUI are rising. Therefore safe and effective novel treatments are needed. Stem cell based therapies may tackle this problem.

## References

[1] Subak L.L., Brubaker L., Chai T.C., Creasman J.M., Diokno A.C., Goode P.S., Kraus S.R., Kusek J.W., Leng W.W., Lukacz E.S. (2008). High costs of urinary incontinence among women electing surgery to treat stress incontinence. Obstet. Gynecol..

[2] Markland A.D., Goode P.S., Redden D.T., Borrud L.G., Burgio K.L. (2010). Prevalence of urinary incontinence in men: Results from the national health and nutrition examination survey. J. Urol..

[3] Delancey J.O. (2010). Why do women have stress urinary incontinence?. Neurourol. Urodyn..

[4] Chermansky C.J., Winters J.C. (2012). Complications of vaginal mesh surgery. Curr. Opin. Urol..

[5] Kerr L.A. (2005). Bulking agents in the treatment of stress urinary incontinence: History, outcomes, patient populations, and reimbursement profile. Rev. Urol..

[6] Scott F.B., Bradley W.E., Timm G.W. (1973). Treatment of urinary incontinence by implantable prosthetic sphincter. Urology.

[7] Vakalopoulos I., Kampantais S., Laskaridis L., Chachopoulos V., Koptsis M., Toutziaris C. (2012). New artificial urinary sphincter devices in the treatment of male iatrogenic incontinence. Adv. Urol..

[8] Dissaranan C., Cruz M.A., Couri B.M., Goldman H.B., Damaser M.S. (2011). Stem cell therapy for incontinence: Where are we now? What is the realistic potential?. Curr. Urol. Rep..

[9] Staack A., Rodriguez L.V. (2011). Stem cells for the treatment of urinary incontinence. Curr. Urol. Rep..

[10] Wang H.J., Chuang Y.C., Chancellor M.B. (2011). Development of cellular therapy for the treatment of stress urinary incontinence. Int. Urogynecol. J..

[11] Cannon T.W., Lee J.Y., Somogyi G., Pruchnic R., Smith C.P., Huard J., Chancellor M.B. (2003). Improved sphincter contractility after allogenic muscle-derived progenitor cell injection into the denervated rat urethra. Urology.

[12] Kwon D., Kim Y., Pruchnic R., Jankowski R., Usiene I., de Miguel F., Huard J., Chancellor M.B. (2006). Periurethral cellular injection: Comparison of muscle-derived progenitor cells and fibroblasts with regard to efficacy and tissue contractility in an animal model of stress urinary incontinence. Urology.

[13] Praud C., Sebe P., Biérinx A.S., Sebille A. (2007). Improvement of urethral sphincter deficiency in female rats following autologous skeletal muscle myoblasts grafting. Cell Transplant..

[14] Fu Q., Song X.F., Liao G.L., Deng C.L., Cui L. (2010). Myoblasts differentiated from adipose-derived stem cells to treat stress urinary incontinence. Urology.

[15] Lin G., Wang G., Banie L., Ning H., Shindel A.W., Fandel T.M., Lue T.F., Lin C.S. (2010). Treatment of stress urinary incontinence with adipose tissue-derived stem cells. Cytotherapy.

[16] Nitta M., Tamaki T., Tono K., Okada Y., Masuda M., Akatsuka A., Hoshi A., Usui Y., Terachi T. (2010). Reconstitution of experimental neurogenic bladder dysfunction using skeletal muscle-derived multipotent stem cells. Transplantation.

[17] Goldman H.B., Sievert K.D., Damaser M.S. (2012). Will we ever use stem cells for the treatment of SUI? ICI-RS 2011. Neurourol. Urodyn..

[18] Kim J.H., Lee S.-R., Song Y.S., Lee H.J. (2013). Stem cell therapy in bladder dysfunction: Where are we? And where do we have to go?. Biomed. Res. Int..

[19] Strasser H., Marksteiner R., Margreiter E., Pinggera G.M., Mitterberger M., Frauscher F., Ulmer H., Fussenegger M., Kofler K., Bartsch G. (2007). Autologous myoblasts and fibroblasts *versus* collagen for treatment of stress urinary incontinence in women: A randomised controlled trial. Lancet.

[20] Strasser H., Tiefenthaler M., Steinlechner M., Bartsch G., Konwalinka G. (1999). Urinary incontinence in the elderly and age-dependent apoptosis of rhabdosphincter cells. Lancet.

[21] Carr L.K., Steele D., Steele S., Wagner D., Pruchnic R., Jankowski R., Erickson J., Huard J., Chancellor M.B. (2008). 1-Year follow-up of autologous muscle-derived stem cell injection pilot study to treat stress urinary incontinence. Int. Urogynecol. J. Pelvic Floor Dysfunct..

[22] Kajbafzadeh A.M., Elmi A., Payabvash S., Salmasi A.H., Saeedi P., Mohamadkhani A., Sadeghi Z., Nikfarjam L. (2008). Transurethral autologous myoblast injection for treatment of urinary incontinence in children with classic bladder exstrophy. J. Urol..

[23] Mitterberger M., Marksteiner R., Margreiter E., Pinggera G.M., Frauscher F., Ulmer H., Fussenegger M., Bartsch G., Strasser H. (2008). Myoblast and fibroblast therapy for post-prostatectomy urinary incontinence: 1-Year followup of 63 patients. J. Urol..

[24] Yamamoto T., Gotoh M., Hattori R., Toriyama K., Kamei Y., Iwaguro H., Matsukawa Y., Funahashi Y. (2010). Periurethral injection of autologous adipose-derived stem cells for the treatment of stress urinary incontinence in patients undergoing radical prostatectomy: Report of two initial cases. Int. J. Urol..

[25] Gerullis H., Eimer C., Georgas E., Homburger M., El-Baz A.G., Wishahi M., Borós M., Ecke T.H., Otto T. (2012). Muscle-derived cells for treatment of iatrogenic sphincter damage and urinary incontinence in men. Sci. World J..

[26] Garcia-Arranz M., Gregorio S.A. (2012). Phase II Clinical Trial to Study Feasibility And Safety of the Expanded Autologous MSC Derived from Adipose Tissue for the Local Feminine Stress Urinary Incontinence. http://clinicaltrials.gov/.

[27] Kleinert S., Horton R. (2008). Retraction—Autologous myoblasts and fibroblasts *versus* collagen [corrected] for treatment of stress urinary incontinence in women: A [corrected] randomised controlled trial. Lancet.

[28] Friedenstein A.J., Gorskaja J.F., Kulagina N.N. (1976). Fibroblast precursors in normal and irradiated mouse hematopoietic organs. Exp. Hematol..

[29] Ashton B.A., Allen T.D., Howlett C.R., Eaglesom C.C., Hattori A., Owen M. (1980). Formation of bone and cartilage by marrow stromal cells in diffusion chambers *in vivo*. Clin. Orthop. Relat. Res..

[30] Bab I., Ashton B.A., Gazit D., Marx G., Williamson M.C., Owen M.E. (1986). Kinetics and differentiation of marrow stromal cells in diffusion chambers *in vivo*. J. Cell Sci..

[31] Friedenstein A.J., Chailakhyan C.A., Gerasimov U.V. (1987). Bone marrow osteogenic stem cells: *In vitro* cultivation and transplantation in diffusion chambers. Cell Tissue Kinet..

[32] Caplan A.I. (1991). Mesenchymal stem cells. J. Orthop. Res..

[33] Pittenger M.F., Mackay A.M., Beck S.C., Jaiswal R.K., Douglas R., Mosca J.D., Moorman M.A., Simonetti D.W., Craig S., Marshak D.R. (1999). Multilineage potential of adult human mesenchymal stem cells. Science.

[34] Zannettino A.C.W., Paton S., Arthur A., Khor F., Itescu S., Gimble J.M., Gronthos S. (2008). Multipotential human adipose-derived stromal stem cells exhibit a perivascular phenotype *in vitro* and *in vivo*. J. Cell Physiol..

[35] Parolini O., Alviano F., Bergwerf I., Boraschi D., de Bari C., de Waele P., Dominici M., Evangelista M., Falk W., Hennerbichler S., Hess D.C. (2010). Toward cell therapy using placenta-derived cells: Disease mechanisms, cell biology, preclinical studies, and regulatory aspects at the round table. Stem Cells Dev..

[36] Covas D.T., Panepucci R.A., Fontes A.M., Silva W.A., Orellana M.D., Freitas M.C., Neder L., Santos A.R., Peres L.C., Jamur M.C., Zago M.A. (2008). Multipotent mesenchymal stromal cells obtained from diverse human tissues share functional properties and gene-expression profile with CD146+ perivascular cells and fibroblasts. Exp. Hematol..

[37] Crisan M., Yap S., Casteilla L., Chen C.W., Corselli M., Park T.S., Andriolo G., Sun B., Zheng B., Zhang L. (2008). A perivascular origin for mesenchymal stem cells in multiple human organs. Cell Stem Cell.

[38] Aggarwal S., Pittenger M.F. (2005). Human mesenchymal stem cells modulate allogeneic immune cell responses. Blood.

[39] Puissant B., Barreau C., Bourin P., Clavel C., Corre J., Bousquet C., Taureau C., Cousin B., Abbal M., Laharrague P. (2005). Immunomodulatory effect of human adipose tissue-derived adult stem cells: Comparison with bone marrow mesenchymal stem cells. Br. J. Haematol..

[40] Sudres M., Norol F., Trenado A., Grégoire S., Charlotte F., Levacher B., Lataillade J.J., Bourin P., Holy X., Vernant J.P. (2006). Bone marrow mesenchymal stem cells suppress lymphocyte proliferation *in vitro* but fail to prevent graft-versus-host disease in mice. J. Immunol..

[41] Jang M.J., Kim H.S., Lee H.G., Kim G.J., Jeon H.G., Shin H.S., Chang S.K., Hur G.H., Chong S.Y., Oh D., Chung H.M. (2013). Placenta-derived mesenchymal stem cells have an immunomodulatory effect that can control acute graft-versus-host disease in mice. Acta Haematol..

[42] Chen L., Tredget E.E., Wu P.Y.G., Wu Y. (2008). Paracrine factors of mesenchymal stem cells recruit macrophages and endothelial lineage cells and enhance wound healing. PLoS One.

[43] Salgado A.J., Reis R.L., Sousa N.J., Gimble J.M. (2010). Adipose tissue derived stem cells secretome: Soluble factors and their roles in regenerative medicine. Curr. Stem Cell Res. Ther..

[44] Minguell J.J., Erices A. (2006). Mesenchymal stem cells and the treatment of cardiac disease. Exp. Biol. Med..

[45] Rackwitz L., Eden L., Reppenhagen S., Reichert J.C., Jakob F., Walles H., Pullig O., Tuan R.S., Rudert M., Nöth U. (2012). Stem cell- and growth factor-based regenerative therapies for avascular necrosis of the femoral head. Stem Cell Res. Ther..

[46] Johnson K., Zhu S., Tremblay M.S., Payette J.N., Wang J., Bouchez C., Meeusen S., Althage A., Cho C.Y., Wu X., Schultz P.J. (2012). A stem cell-based approach to cartilage repair. Science.

[47] Hughey C.C., Johnsen V.L., Ma L., James F.D., Young P.P., Wasserman D.H., Rottman J.N., Hittel D.S., Shearer J. (2012). Mesenchymal stem cell transplantation for the infarcted heart: A role in minimizing abnormalities in cardiac-specific energy metabolism. Am. J. Physiol. Endocrinol. Metab..

[48] Quevedo H.C., Hatzistergos K.E., Oskouei B.N., Feigenbaum G.S., Rodriguez J.E., Valdes D., Pattany P.M., Zambrano J.P., Hu Q., McNiece I. (2009). Allogeneic mesenchymal stem cells restore cardiac function in chronic ischemic cardiomyopathy via trilineage differentiating capacity. Proc. Natl. Acad. Sci. USA.

[49] Gu W., Zhang F., Xue Q., Ma Z., Lu P., Yu B. (2010). Transplantation of bone marrow mesenchymal stem cells reduces lesion volume and induces axonal regrowth of injured spinal cord. Neuropathology.

[50] Fukuda K. (2003). Use of adult marrow mesenchymal stem cells for regeneration of cardiomyocytes. Bone Marrow Transplant..

[51] Kanematsu A., Yamamoto S., Iwai-Kanai E., Kanatani I., Imamura M., Adam R.M., Tabata Y., Ogawa O. (2005). Induction of smooth muscle cell-like phenotype in marrow-derived cells among regenerating urinary bladder smooth muscle cells. Am. J. Pathol..

[52] Drost A.C., Weng S., Feil G., Schäfer J., Baumann S., Kanz L., Sievert K.D., Stenzl A., Möhle R. (2009). *In vitro* myogenic differentiation of human bone marrow-derived mesenchymal stem cells as a potential treatment for urethral sphincter muscle repair. Ann. N. Y. Acad. Sci..

[53] Kinebuchi Y., Aizawa N., Imamura T., Ishizuka O., Igawa Y., Nishizawa O. (2010). Autologous bone-marrow-derived mesenchymal stem cell transplantation into injured rat urethral sphincter. Int. J. Urol..

[54] Gunetti M., Tomasi S., Giammò A., Boido M., Rustichelli D., Mareschi K., Errichiello E., Parola M., Ferrero I., Fagioli F., Vercelli A. (2012). Myogenic potential of whole bone marrow mesenchymal stem cells *in vitro* and *in vivo* for usage in urinary incontinence. PLoS One.

[55] Dominici M., Le Blanc K., Mueller I., Slaper-Cortenbach I., Marini F., Krause D., Deans R., Keating A., Prockop D.J., Horwitz E. (2006). Minimal criteria for defining multipotent mesenchymal stromal cells. The International Society for Cellular Therapy position statement. Cytotherapy.

[56] Zuk P.A., Zhu M., Mizuno H., Huang J., Futrell J.W., Katz A.J., Benhaim P., Lorenz H.P., Hedrick M.H. (2001). Multilineage cells from human adipose tissue: Implications for cell-based therapies. Tissue Eng..

[57] Lang R., Liu G., Shi Y., Bharadwaj S., Leng X., Zhou X., Liu H., Atala A., Zhang Y. (2013). Self-renewal and differentiation capacity of urine-derived stem cells after urine preservation for 24 hours. PLoS One.

[58] Liu G., Pareta R.A., Wu R., Shi Y., Zhou X., Liu H., Deng C., Sun X., Atala A., Opara E.C., Zhang Y. (2013). Skeletal myogenic differentiation of urine-derived stem cells and angiogenesis using microbeads loaded with growth factors. Biomaterials.

[59] Montarras D., Morgan J., Collins C., Relaix F., Zaffran S., Cumano A., Partridge T., Buckingham M. (2005). Direct isolation of satellite cells for skeletal muscle regeneration. Science.

[60] Chapman M.R., Balakrishnan K.R., Li J., Conboy M.J., Huang H., Mohanty S.K., Jabart E., Hack J., Conboy I.M., Sohn L.L. (2013). Sorting single satellite cells from individual myofibers reveals heterogeneity in cell-surface markers and myogenic capacity. Integr. Biol. (Camb.).

[61] Muller I., Vaegler M., Holzwarth C., Tzaribatchev N., Pfister S.M., Schütt B., Reize P., Greil J., Handgretinger R., Rudert M. (2008). Secretion of angiogenic proteins by human multipotent mesenchymal stromal cells and their clinical potential in the treatment of avascular osteonecrosis. Leukemia.

[62] Felka T., Schäfer R., de Zwart P., Aicher W.K. (2010). Animal serum-free expansion and differentiation of human mesenchymal stromal cells. Cytotherapy.

[63] Persson J., Wolner-Hanssen P., Rydhstroem H. (2000). Obstetric risk factors for stress urinary incontinence: A population-based study. Obstet. Gynecol..

[64] Pilz G.A., Ulrich C., Ruh M., Abele H., Schäfer R., Kluba T., Bühring H.J., Rolauffs B., Aicher W.K. (2011). Human term placenta-derived mesenchymal stromal cells are less prone to osteogenic differentiation than bone marrow-derived mesenchymal stromal cells. Stem Cells Dev..

[65] Ulrich C., Rolauffs B., Abele H., Bonin M., Nieselt K., Hart M.L., Aicher W.K. (2013). Low osteogenic differentiation potential of placenta-derived mesenchymal stromal cells correlates with low expression of the transcription factors Runx2 and Twist2. Stem Cells Dev..

[66] Zhang Y., McNeill E., Tian H., Soker S., Andersson K.E., Yoo J.J., Atala A. (2008). Urine derived cells are a potential source for urological tissue reconstruction. J. Urol..

[67] Bodin A., Bharadwaj S., Wu S., Gatenholm P., Atala A., Zhang Y. (2010). Tissue-engineered conduit using urine-derived stem cells seeded bacterial cellulose polymer in urinary reconstruction and diversion. Biomaterials.

[68] Wu S., Wang Z., Bharadwaj S., Hodges S.J., Atala A., Zhang Y. (2011). Implantation of autologous urine derived stem cells expressing vascular endothelial growth factor for potential use in genitourinary reconstruction. J. Urol..

[69] Wu S., Liu Y., Bharadwaj S., Atala A., Zhang Y. (2011). Human urine-derived stem cells seeded in a modified 3D porous small intestinal submucosa scaffold for urethral tissue engineering. Biomaterials.

[70] Phinney D.G., Prockop D.J. (2007). Concise review: Mesenchymal stem/multipotent stromal cells: The state of transdifferentiation and modes of tissue repair—Current views. Stem Cells.

[71] Wallner C., Dabhoiwala N.F., DeRuiter M.C., Lamers W.H. (2009). The anatomical components of urinary continence. Eur. Urol..

[72] Gang E.J., Jeong J.A., Hong S.H., Hwang S.H., Kim S.W., Yang I.H., Ahn C., Han H., Kim H. (2004). Skeletal myogenic differentiation of mesenchymal stem cells isolated from human umbilical cord blood. Stem Cells.

[73] Dezawa M., Ishikawa H., Itokazu Y., Yoshihara T., Hoshino M., Takeda S., Ide C., Nabeshima Y. (2005). Bone marrow stromal cells generate muscle cells and repair muscle degeneration. Science.

[74] Warren L., Manos P.D., Ahfeldt T., Loh Y.H., Li H., Lau F., Ebina W., Mandal P.K., Smith Z.D., Meissner A. (2010). Highly efficient reprogramming to pluripotency and directed differentiation of human cells with synthetic modified mRNA. Cell Stem Cell.

[75] Mauro A. (1961). Satellite cells of skeletal muscle fibers. J. Biophys. Biochem. Cytol..

[76] Wagers A.J., Conboy I.M. (2005). Cellular and molecular signatures of muscle regeneration: Current concepts and controversies in adult myogenesis. Cell.

[77] Chancellor M.B., Yokoyama T., Tirney S., Mattes C.E., Ozawa H., Yoshimura N., de Groat W.C., Huard J. (2000). Preliminary results of myoblast injection into the urethra and bladder wall: A possible method for the treatment of stress urinary incontinence and impaired detrusor contractility. Neurourol. Urodyn..

[78] Yiou R., Lefaucheur J.P., Atala A. (2003). The regeneration process of the striated urethral sphincter involves activation of intrinsic satellite cells. Anat. Embryol. (Berl.).

[79] Kwon D., Minnery B., Kim Y., Kim J.H., de Miguel F., Yoshimura N., Chancellor M.B. (2005). Neurologic recovery and improved detrusor contractility using muscle-derived cells in rat model of unilateral pelvic nerve transection. Urology.

[80] Elmi A., Kajbafzadeh A.M., Tourchi A., Talab S.S., Esfahani S.A. (2011). Safety, efficacy and health related quality of life of autologous myoblast transplantation for treatment of urinary incontinence in children with bladder exstrophy-epispadias complex. J. Urol..

[81] Winkler T., von Roth P., Matziolis G., Mehta M., Perka C., Duda G.N. (2009). Dose-response relationship of mesenchymal stem cell transplantation and functional regeneration after severe skeletal muscle injury in rats. Tissue Eng. A.

[82] Lee J.Y., Cannon T.W., Pruchnic R., Fraser M.O., Huard J., Chancellor M.B. (2003). The effects of periurethral muscle-derived stem cell injection on leak point pressure in a rat model of stress urinary incontinence. Int. Urogynecol. J..

[83] Eberli D., Aboushwareb T., Soker S., Yoo J.J., Atala A. (2012). Muscle precursor cells for the restoration of irreversibly damaged sphincter function. Cell Transplant..

[84] Jiang H.H., Damaser M.S. (2011). Animal models of stress urinary incontinence. Handb. Exp. Pharmacol..

[85] Conway D.A., Kamo I., Yoshimura N., Chancellor M.B., Cannon T.W. (2005). Comparison of leak point pressure methods in an animal model of stress urinary incontinence. Int. Urogynecol. J. Pelvic Floor Dysfunct..

[86] Schultz-Lampel D., Goepel M., Haferkamp A. (2012). Urodynamik.

[87] Rossier A.B., Fam B.A. (1986). 5-Microtransducer catheter in evaluation of neurogenic bladder function. Urology.

[88] Griffiths D. (1985). The pressure within a collapsed tube, with special reference to urethral pressure. Med. Biol..

[89] Hadamard J. (2003). Lectures on Cauchy’s Problem: In Linear Partial Differential Equations.

[90] Bonnet M., Constantinescu A. (2005). Inverse problems in elasticity. Inverse Probl..

[91] Balocco S., Basset O., Courbebaisse G., Boni E., Frangi A.F., Tortoli P., Cachard C. (2010). Estimation of the viscoelastic properties of vessel walls using a computational model and doppler ultrasound. Phys. Med. Biol..

[92] Perego M., Veneziani A., Vergara C. (2011). A variational approach for estimating the compliance of the cardiovascular tissue: An inverse fluidstructure interaction problem. SIAM J. Sci. Comput..

[93] Bertoglio C., Moireau P., Gerbeau J. (2012). Sequential parameter estimation for fluid—Structure problems: Application to hemodynamics. Int. J. Numerical. Meth. Biomed. Eng..

[94] Norton P., Brubaker L. (2006). Urinary incontinence in women. Lancet.

[95] Schafer R., Bantleon R., Kehlbach R., Siegel G., Wiskirchen J., Wolburg H., Kluba T., Eibofner F., Northoff H., Claussen C.D., Schlemmer H.P. (2010). Functional investigations on human mesenchymal stem cells exposed to magnetic fields and labeled with clinically approved iron nanoparticles. BMC Cell Biol..

[96] Guan X., Delo D.M., Atala A., Soker S. (2011). *In vitro* cardiomyogenic potential of human amniotic fluid stem cells. J. Tissue Eng. Regen. Med..

[97] Karnoub A.E., Dash A.B., Vo A.P., Sullivan A., Brooks M.W., Bell G.W., Richardson A.L., Polyak K., Tubo R., Weinberg R.A. (2007). Mesenchymal stem cells within tumour stroma promote breast cancer metastasis. Nature.

[98] Pearson T., Greiner D.L., Shultz L.D. (2008). Humanized SCID mouse models for biomedical research. Curr. Top. Microbiol. Immunol..

[99] Caplan A.I., Correa D. (2011). The MSC: An injury drugstore. Cell Stem Cell.

